# Correlation between human health and reactive oxygen species produced in blood: a long-term chemiluminescence and fluorescence analysis

**DOI:** 10.1038/s41598-021-93887-1

**Published:** 2021-07-15

**Authors:** Kimiko Kazumura, Kozo Takeuchi, Yukiko Hatano, Akiko Hara, Toshiyuki Miwa, Masaki Hattori, Fusanori Kondo, Naokazu Morishita, Hiroshi Tsuchiya, Toshihiko Osawa

**Affiliations:** 1 Central Research Laboratory, Hamamatsu Photonics K.K., Shizuoka, Japan; 2 Global Strategic Challenge Center, Hamamatsu Photonics K.K., Shizuoka, Japan; 3 Electron Tube Division, Hamamatsu Photonics K.K., Shizuoka, Japan; 4 Department of Health and Nutrition, Faculty of Psychological and Physical Science, Aichi Gakuin University, Aichi, Japan

**Keywords:** Biomarkers, Health care

## Abstract

The previous slide-glass type system could simultaneously detect reactive and highly reactive oxygen species, i.e., superoxide radicals (O_2_^−·^) and hypochlorite ions (OCl^−^) elicited from leucocytes in sample blood, but had some drawbacks, i.e., signal noise from air-flow stirring, potential biohazard risks, etc. because of open samples placed on a slide glass. We overcame these drawbacks by adopting a fluidic-chip container in a new system, which resulted in higher sensitivity and more stable measurements. Using the new system, we conducted a pilot study on nominally healthy volunteers to find whether or not the monitored activities of leukocytes can distinguish more or less unhealthy conditions from healthy ones. At first, healthy volunteers of both genders and of various ages showed that the fluctuation magnitudes (%) of O_2_^−·^ and OCl^−^ were nearly similar to each other and to that of the neutrophil count fluctuation. These parameters sometimes exceeded the healthy fluctuation range. By comparing these large fluctuations with the data of an inflammation marker C-reactive protein (CRP), the neutrophil count fluctuation and the timings/symptoms of abnormalities found in questionnaire, we could gain information suggesting the factors causing the large fluctuations. The new system could detect bodily abnormalities earlier than CRP or self-aware symptoms.

## Introduction

Helmut Sies introduced the concept of “oxidative stress” in 1985, inspired by Hans Selye’s stress theory^1^. The definition was updated in 2015 as “redox signaling/control disrupting and molecular damaging imbalance between oxidants and antioxidants”^2^. Since the introduction of the concept, many scientists began to study oxidants, free radicals and antioxidants in biological processes, which revealed biological roles of reactive oxygen species (ROS) including superoxide radicals (O_2_^−·^) and hydrogen peroxide, and highly reactive oxygen species (hROS) including hypochlorite ions (OCl^−^). Therefore, intensity-based classification and mechanism-based sub-categorization of oxidative stress would be required^2,3^. According to Lushchak, oxidative stress can be restated as imbalance between generation and elimination of ROS in biological systems^3^. In recent years, involvements of ROS in various diseases have increasingly become evident^4–6^.

A central player in the innate immune responses, i.e., white blood cells (WBC), is known to produce a large amount of ROS enzymatically^4–7^. Therefore, development of technologies for monitoring ROS and hROS produced by these cells would contribute to study oxidative stress.

Previously we developed a simultaneous chemiluminescence (CL) and fluorescence (FL) monitoring system CFL-C2000^8^. By improving the system, we have recently developed CFL-P2200^9^ and demonstrated that it can semi-automatically detect ROS and hROS, i.e., O_2_^−·^ and OCl^−^ elicited in a small amount of blood samples by using CL and FL detecting reagents. This newer system was recently applied to studying the correlative relationships between O_2_^−·^/OCl^−^ and diseases, such as Alzheimer's disease^10^, arteriosclerosis^11^, and hypertension^12^ with the rodent models. Other than these applications, it can principally be used for any other colored liquids because the effect of light absorption was effectively minimized.

Although the newer CFL-P2200 has many advantages over the older CFL-C2000 or other conventional methods, we noticed that it is still in need of more improvements for measuring blood samples easily and reliably. With CFL-P2200, samples were placed on a dedicated glass slide and were stirred by air flows. First and the most serious issue is that it may pose potential biohazard risks because the blood samples placed on a glass slide are not covered and constantly exposed to the system and its operators. Secondly, air flow agitations of the sample surfaces cause optical data fluctuations, which have to be removed by offline data averaging and/or other analyzing techniques. Besides, fine-tuning of the air flows (amounts, timing and directions) requires some practice and training.

To solve these problems, a special thin fluidic chip, which is wider and thinner than the conventional optical cuvette has been developed. A stirrer bar is designed to be included in the chip. The chip is placed vertically and the high-sensitive optical configuration of CFL-P2200 was adapted accordingly. Vertical setting of the chip is more advantageous than the horizontal setting in the sense that the latter would require a special structure for introducing samples, as exemplified by an electrophoresis chip^13^.

Here we report that, through these improvements, the newest system is not only more suitable for blood samples, but also O_2_^−·^/OCl^−^ can be measured more sensitively. Further, this system can achieve more stable data for a much longer period of time than CFL-P2200. Using the new system, we conducted an initial pilot study on nominally healthy volunteers. More precisely, all volunteers cleared annual health checkups and are considered healthy in that sense. But all of them experienced periods of more or less unhealthy conditions from time to time. Therefore, one of the purposes of this study is to find about whether or not the monitored activities of leukocytes can provide information to distinguish more or less unhealthy conditions from healthy ones. Since this approach to measuring leukocyte activity has not been available nor attempted in all conventional methods, we had to start collecting basic data on healthy subjects at first. For over several months, we monitored O_2_^−·^/OCl^−^ in blood taken from six healthy volunteers. Each volunteer’s day-to-day O_2_^−·^/OCl^−^ productions were found to be fluctuated uniquely and the production dynamics varied among individuals. Comparison of O_2_^−·^/OCl^−^ fluctuations with the neutrophil count, levels of a conventional inflammation marker and the physical state variability of volunteers deduced from reported questionnaires revealed many notable findings. We also report a new method to analyze O_2_^−·^/OCl^−^ production capacities in blood.

## Materials and methods

### Reagents

Phorbol 12-myristate 13-acetate (PMA) and dimethyl sulfoxide (DMSO) were purchased from Sigma-Aldrich Japan (Tokyo, Japan). 2-Methyl-6-(4-methoxyphenyl)-3,7-dihydroimidazo [1,2-a] pyrazin-3-one hydrochloride (MCLA) was purchased from Tokyo Kasei (Tokyo, Japan). Aminophenyl fluorescein (APF) was purchased from GORYO Chemical (Sapporo, Japan).

PMA (0.1 mM) was dissolved into DMSO and stored at − 80 °C as PMA stock solution. It was diluted 1:20 (v/v) with Ringer–Hepes buffer (RH buffer: 154 mM NaCl, 5.6 mM KCl, and 10 mM Hepes, pH7.4) just prior to use. MCLA was dissolved in Milli-Q water and its concentration was adjusted according to the molar extinction coefficient after filtration.

### The fluidic-chip type sample container

**Figure 1:**
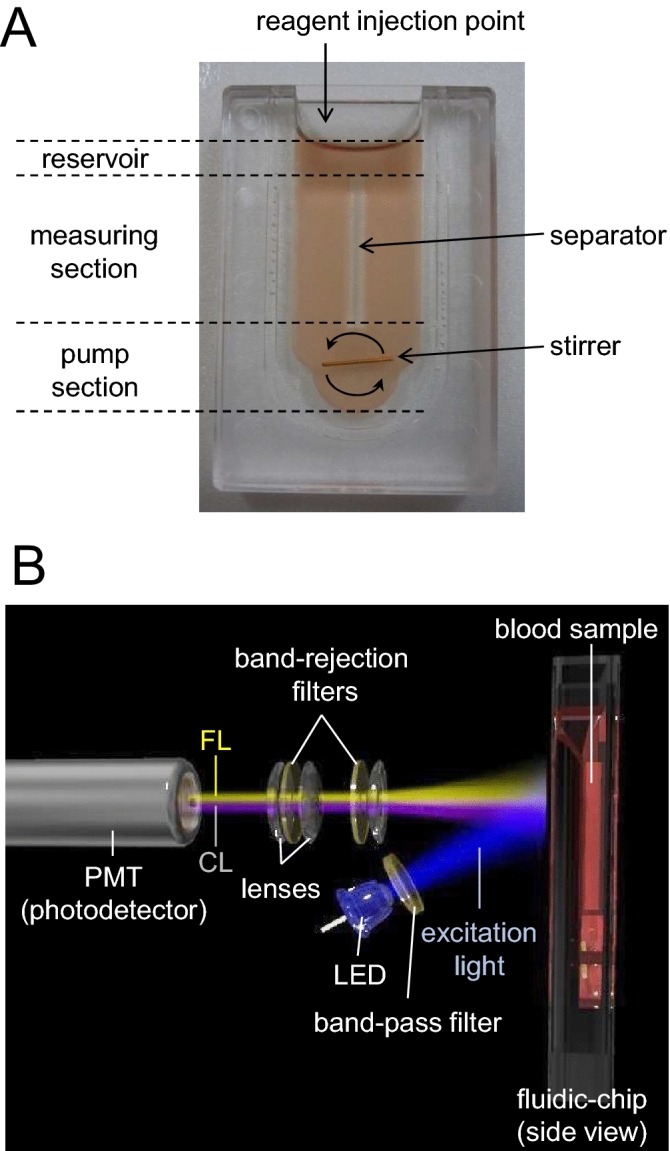
The fluidic-chip and optical configuration for the new system. (**A**) A picture showing the structure of the fluidic-chip (outer dimensions: width 28 mm, thickness 6 mm and height 41 mm; inner dimensions: width 13.4 mm, thickness 2 mm and maximum depth 33.5 mm; internal sample volume: 750 µL). (**B**) A schematic diagram of the optical configuration for CFL-H2200 and -H2400 models. Detailed explanations are described in “Materials and methods”. This 3D visualization in (**B**) was created with the help of Dr. Hiroshi Satozono (Hamamatsu Photonics K.K.).

The newly developed thin fluidic-chip dedicated to CFL-H2200 and -H2400 models is shown in Fig. 1, Fig. S1A and Table 1. The optical path length is set to 2 mm, which gives enough signals while effectively minimizing the influence of light scattering by blood components and light absorption of red blood cells. The chip is composed of three sections: the pump section, the measuring section, and the reservoir (Fig. 1A). The dedicated stirrer, an inexpensive metal pin covered with polytetrafluoroethylene (PTFE) tube, is put in the pump section. The separator placed in the center of the chip enabled to stir the solution uniformly (Fig. 1A). The pump section and the optically measuring section were also separated for minimizing the effects of the stirring process during measurement. The upper side of the chip is wide open to allow reagents, solutions and a stirrer to be easily introduced.

**Table 1:** A list of simultaneous CL and FL monitoring systems so far developed.

System	Key reference	Sample container	Stirring	Channel(s)	Sample volume (µL)	Size (mm)	Weight (kg)
CFL-C2000	Kazumura et al. (2013)	Cuvette (commercially available)	Stirrer (commercially available)	1	1500	W160 × D250 × H90	2.6
CFL-P2200	Kazumura et al. (2018)	Glass slide (custom-made)	Air flows (newly developed)	2	500	W213 × D233 × H224	12.3
CFL-H2200	This study	Fluidic-chip (newly developed)	Dedicated stirrer (newly developed)	2	750	W123 × D232 × H204	3.4
CFL-H2400	This study	Fluidic-chip (newly developed)	Dedicated stirrer (newly developed)	4	750	W213 × D232 × H204	7.2

CFL-H2200/H2400 achieved the goal which is smaller, lighter, and more energy-efficient than the glass slide-type, CFL-P2200.

### Optical configuration of the system

The optical configuration of the newest CFL-H2200 and -H2400 prototype models (Hamamatsu Photonics K.K., Hamamatsu, Japan) is basically the same as CFL-P2200 (Fig. 1B, Fig. S2B,C). A single photomultiplier tube (PMT; H10682-210, Hamamatsu Photonics K.K.) is used for the detection of CL and FL. In order to efficiently focus the surface-reflected FL suitable for FL detection from colored samples, light emitting diode (LED) as the excitation light was placed on the PMT side. The reason is that the same-side illumination is very effective to cut off excitation light from FL signals, while, in the transmission configuration of FL detection the light signal contains considerable amount of excitation light which is very hard to eliminate through optical filters because the excitation light intensity is very high and considerably higher than the FL signal itself. The wavelength of the excitation light (band-pass filter 480 nm, FWHM 10 nm) is optimized for the FL reagent APF (Ex-Max 490 nm). A light-collecting lens and optical filter set is placed in front of the PMT. The band-rejection filters in the set are optimized for efficiently blocking excitation light and transmitting MCLA-derived CL (Em-Max 465 nm) and APF-derived FL (Em-Max 515 nm). Separation of CL and FL submitted to a single PMT was attained by repeating on/off of the excitation lights at a high-enough speed^8,9,14^.

### Features of CFL-H2200 and -H2400 models

CFL-H2200 has two measuring channels, while CFL-H2400 has four (i.e., CFL-H2200 can simultaneously set and measure two samples, CFL-H2400; four samples, Fig. S1A,B). Other specifications are basically common (Table 1). In order to keep compatibilities among different channels/systems, the sensitivity of each PMT channel was corrected by the standardized illuminant having an identical shape as the above mentioned fluidic-chip. This illuminant is designed to emit the same level of light as blood samples.

### Whole blood collection

Blood samples were self-collected from fingertips of volunteers by using a lancet (Becton, Dickinson and Company, Franklin Lakes, NJ, USA or Nipro, Osaka, Japan). They were preserved in BD Microtainer Tubes (Becton, Dickinson and Company, Franklin Lakes, NJ, USA) whose inside was coated with K_2_EDTA to prevent blood coagulation. They were kept at room temperature and used within 2 h after collection. Informed consent was obtained in every blood collection. All experiments were performed in full compliance with the guidelines of the Research Ethics Committee of the Hamamatsu Photonics K.K. and approved by the Research Ethics Committee of the Hamamatsu Photonics K.K. under the number H-117.

### Blood cell counting and CRP measurement

The blood cell counts and the concentration of C-reactive protein (CRP) in whole blood were determined by Pentra MS CRP (Horiba, Kyoto, Japan) according to the manufacturer’s instruction.

### Assessment of O_2_^−·^ and OCl^−^ generated in blood

O_2_^−·^ is known as the primary ROS^6^ and OCl^−^ **(**hROS) is produced for host defense by WBC. They were elevated by using stimulants and detected as CL of MCLA and FL of APF, respectively. PMA was used for stimulation in this study. The stimulant induced CL and dFL/dt were shown to be proportional to the instantaneous generation of O_2_^−·^ and OCl^−^, respectively^9^. Therefore, for simplicity, we denote these parameters as CL-O_2_^−·^ and FL-OCl^−^ hereafter.

### Monitoring procedure of CL-O_2_^−·^ and FL-OCl^−^

Simultaneous monitoring of CL-O_2_^−·^ and FL-OCl^−^ by CFL-H2200 and -H2400 models was performed with the following procedure (see also Table 2):

1. Introduction of RH buffer containing 0.5 µM MCLA, 10 µM APF and 1 mM CaCl_2_ into the dedicated fluidic-chip.
2. Pre-incubate for 4 min at 37 °C with stirring in the dedicated incubator (Fig. S1C).
3. Add 3 µL of whole blood to the solution.
4. Pre-incubate the chip for 1 min at 37 °C with stirring.
5. Set the fluidic-chip in the system.
6. Pre-incubate another 1 min at 37 °C with stirring in the system.
7. Start measurement and recording of CL-O_2_^−·^ and FL-OCl^−^.
8. Stimulation of the sample by adding PMA automatically at the set timing (controlled by the dedicated measurement software).
9. Data analysis by the dedicated analyzing software, i.e., analysis of CL-O_2_^−·^ and FL-OCl^−*^

**Table 2:** Protocol of the measurement for CL-O_2_^−·^ and FL-OCl^−^ in blood.

Reagent	Stock conc.	Volume (µL)	Final conc.
RH buffer	–	730.5	–
MCLA	50 µM	7.5	0.5 µM
CaCl_2_	100 mM	7.5	1 mM
APF	5 mM	1.5	10 µM
Blood	–	3	1/250
PMA	5 µM	15	0.1 µM

The reaction mixture with the final concentrations presented in the table were directly prepared within the fluidic chip. The procedural protocol is shown as step 1 through 9.

(The incubation time in steps 2, 4, and 6 is required for the sample temperature to reach 37 °C).

^*^The total O_2_^−·^ produced after stimulation is proportional to CL AUC^9,14^, and, since FL is the cumulative amount of OCl^−^, the total OCl^−^ produced after stimulation is equal to FL_MAX_ − FL _BASE POINT_ (‘a−b’ in Fig. S3). FL _BASE POINT_ is the y-value on the horizontal line which passes the rising point of FL-OCl^−^ at the x-value of FL-OCl^−^_MAX_ (blue dotted line in Fig. S3).

### Analysis of day-to-day variation in healthy volunteers

The six subjects, who had no underlying disease in regular health checkups, participated in this study are as follows:

Subject 1) a 35 year-old (y/o) female (n = 21);

Subject 2) a 42 y/o female (n = 24);

Subject 3) a 54 y/o female (n = 63);

Subject 4) a 35 y/o male (n = 39);

Subject 5) a 43 y/o male (n = 22);

Subject 6) a 52 y/o male (n = 25).

Blood were collected in the morning (8:30–10:30) after fasted for more than 10 h for minimizing diet effects. Experiments and questionnaires to the volunteers were performed by non-subjects. The questionnaire includes fasting/sleeping hours and presence or absence of subjective symptoms/medication/doctor's assessments.

## Results

### CL and FL generated in blood measured by using the new fluidic-chip based system

**Figure 2:**
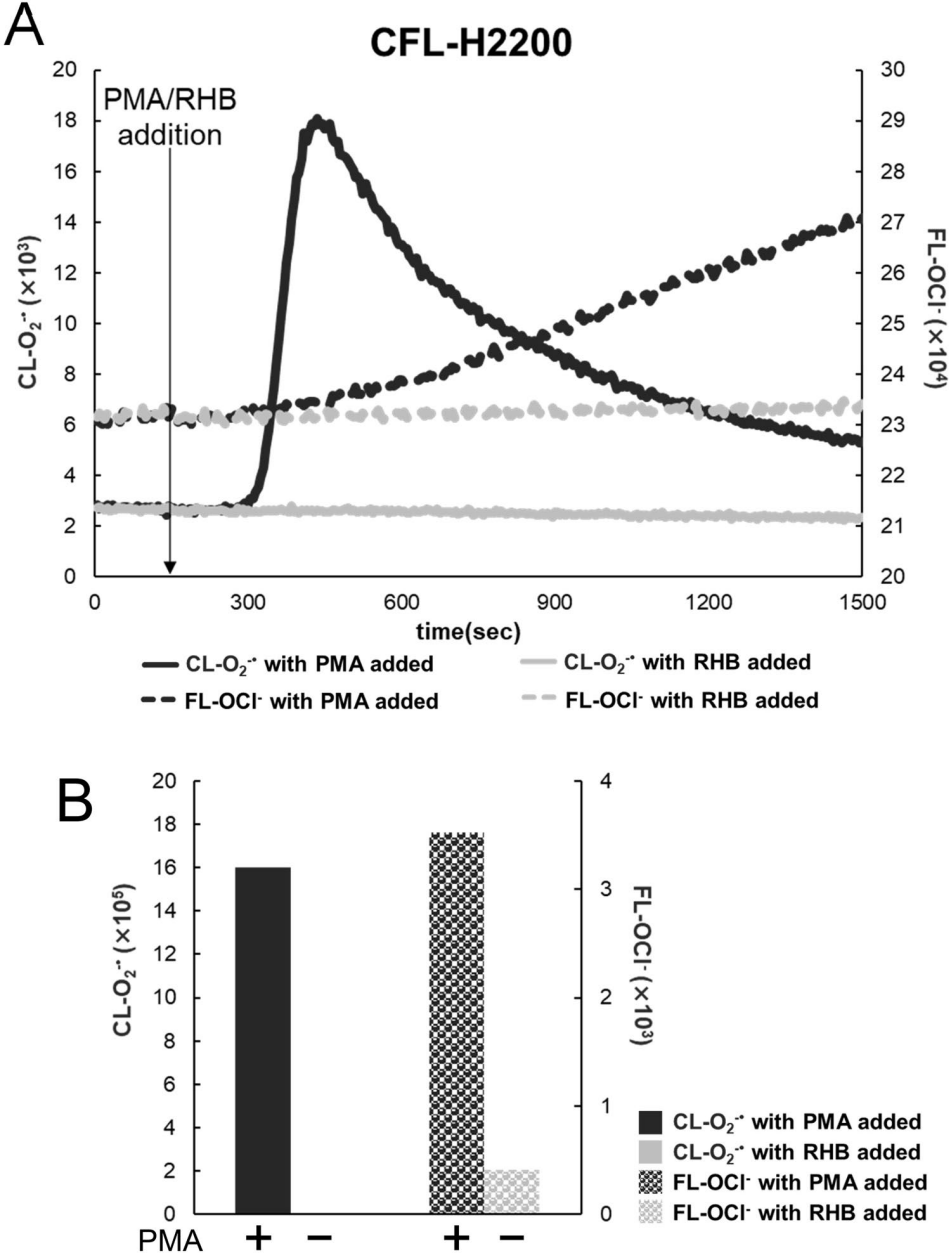
Measurements of CL-O_2_^−·^ and FL-OCl^−^ in blood by the fluidic-chip based system. (**A**) Time courses of CL-O_2_^−·^ (solid lines) and FL-OCl^−^ (dotted lines) in blood obtained by CFL-H2200. The left vertical axis shows CL-O_2_^−·^ intensity; the right vertical axis, FL-OCl^−^ intensity. PMA (dark lines) or RHB (light lines) was added at 150 s (arrow line). The baseline heights of FL-OCl^−^ were adjusted at the same level. (**B**) Baseline-subtracted total amount of CL-O_2_^−·^ and FL-OCl^−^ with and without PMA calculated from the data in (**A**).

With the new fluidic-chip based system (the 2-channel model CFL-H2200), we could obtain larger and more robust O_2_^−·^ and OCl^−^ signals than those obtained with the previous glass slide-type system (CFL-P2200). Figure﻿ 2A shows CL-O_2_^−·^ and FL-OCl^−^ time courses in whole blood with and without the stimulant (PMA). It can be seen that all four time courses are quite similar to those obtained previously^9^. By using the same batch of blood sample, we compared the Fig. 2A time courses with those (Fig. S4A) obtained in the previous system CFL-P2200. The result indicates that the new system gives about 3.9 times higher signals for CL and about 2.8 times higher for FL (Fig. 2B, Fig. S4B). It is also noticeable that the noise levels of the new system were much lower than CFL-P2200.

**Figure 3:**
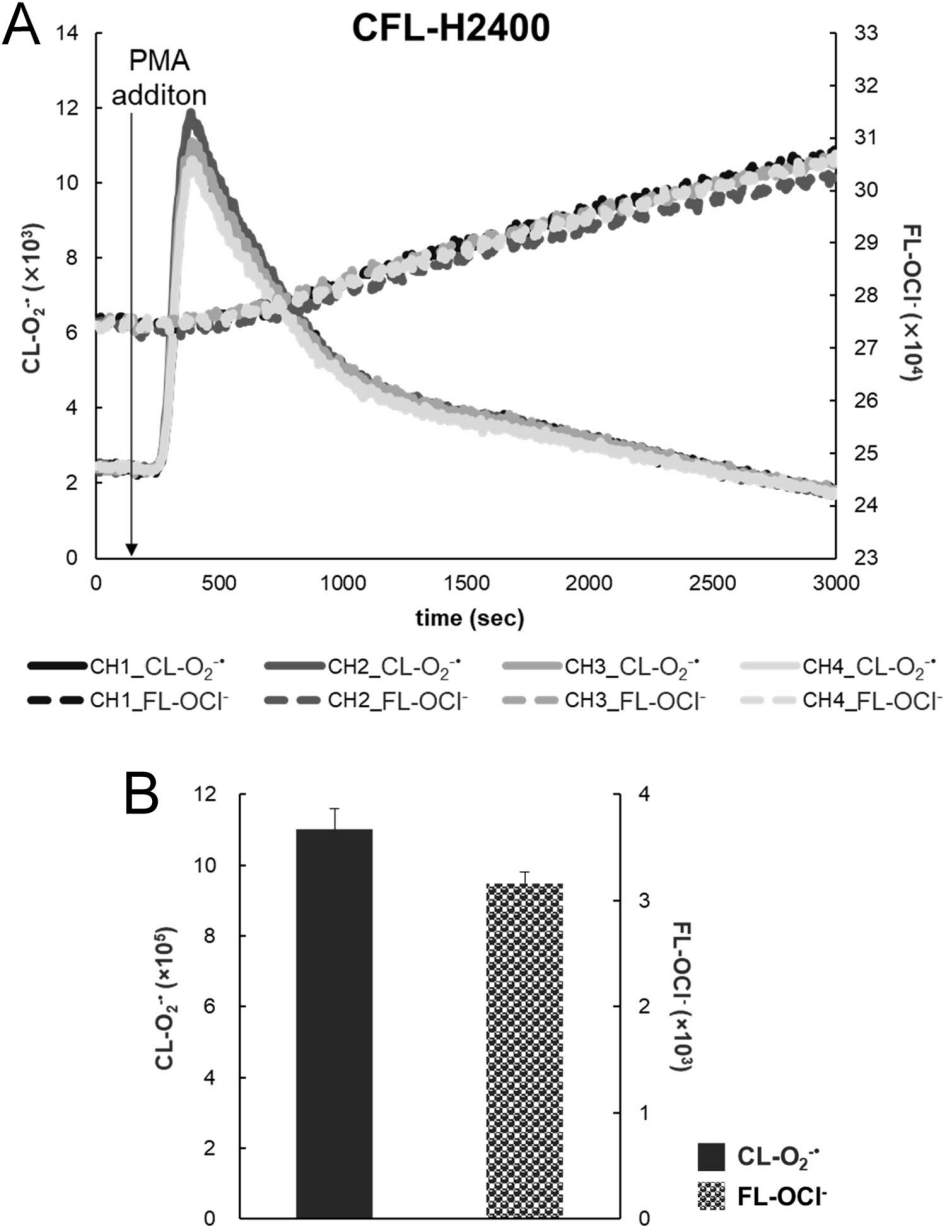
Reproducibility of CL-O_2_^−·^ and FL-OCl^−^ measurements in blood. (**A**) Time courses of CL-O_2_^−·^ (solid lines) and FL-OCl^−^ (dotted lines) in blood. Four channels of CFL-H2400 were used simultaneously. The left vertical axis shows CL-O_2_^−·^ intensity; the right vertical axis, FL-OCl^−^ intensity. PMA was added at 150 s (arrow line). The baseline heights of FL-OCl^−^ were adjusted at the same level. (**B**) Baseline-subtracted total amount of CL-O_2_^−·^ and FL-OCl^−^ calculated from the data in (**A**). Error ratios of the four measurements were 2.62% for the total amount of CL-O_2_^−·^ and 1.74% for that of FL-OCl^−^.

Next, we tested the reproducibility of measurement among the measuring channels equipped with each sample-filled chip by using the 4-channel model (CFL-H2400). Figure 3A shows that all 4-channel data were almost identical to each other. The error ratios among the four channels were 2.62% in CL-O_2_^−·^ and 1.74% in FL-OCl^−^ (Fig. 3B). The error ratios between the models CFL-H2200 and CFL-H2400 were at the same level (data not shown).

### Long term measurements of CL and FL in six healthy volunteers

Since the new system was found to be more sensitive and more stable to measure blood samples for a long time reproducibly, we studied O_2_^−·^ and OCl^−^ signal variations over time in each volunteer and variability of the signals among individuals. As described in “Materials and methods”, blood samples were drawn before breakfast for over several months (for minimizing the effects of food consumption). Six volunteers were chosen from males and females in their 30 s, 40 s and 50 s.

**Figure 4:**
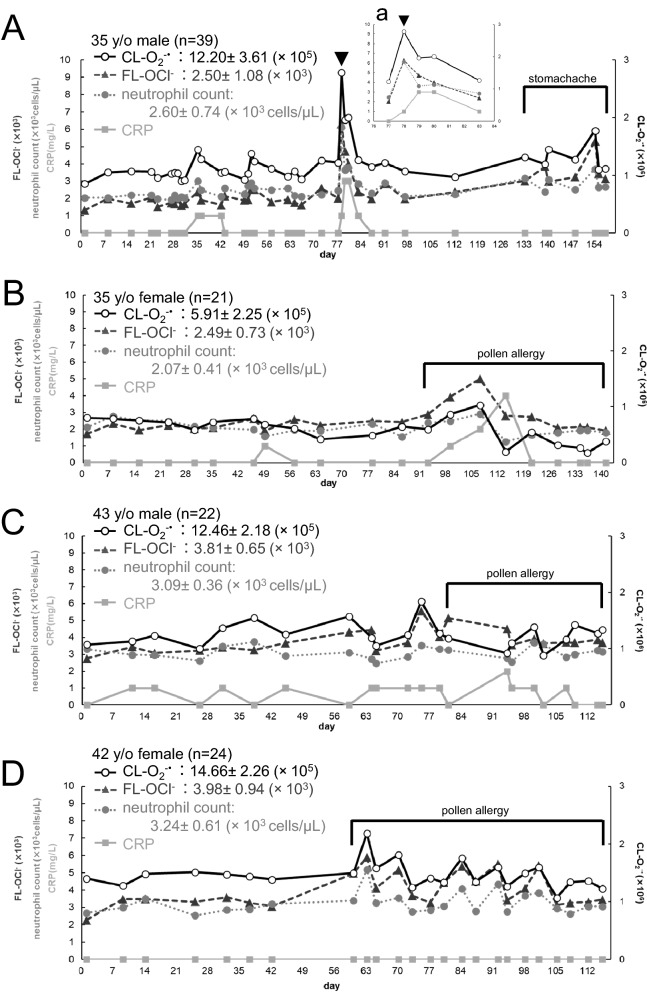

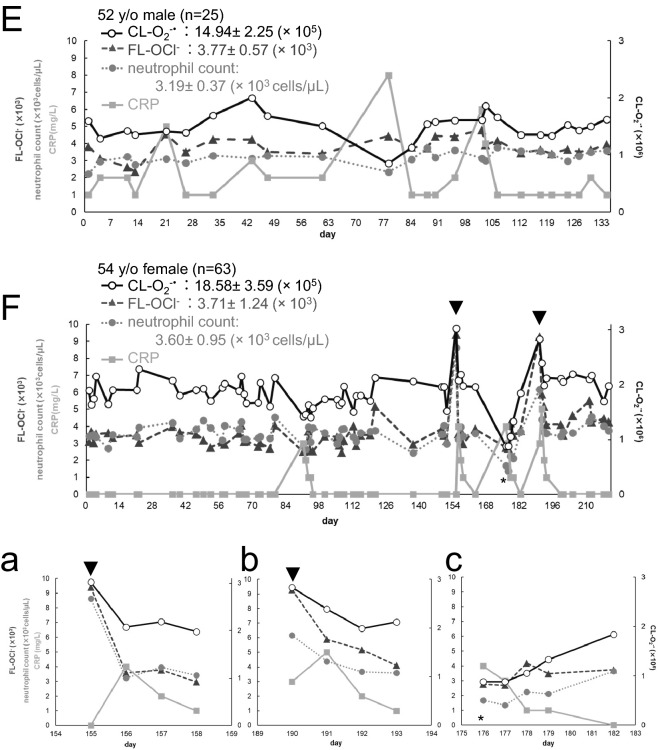
Day-to-day variations of CL-O_2_^−·^ and FL-OCl^−^ in six healthy subjects. Scatter plots showing the day-to-day fluctuations of CL-O_2_^−·^ (○ solid line), FL-OCl^−^ (▲ dashed line), neutrophil counts (● dotted line), and CRP (■ solid line) in six healthy volunteers. The left vertical axes indicate FL-OCl^−^, neutrophil counts and CRP; the right vertical axes, CL-O_2_^−·^. The horizontal axes indicate dates (weekly intervals) during the measurements. The mean values of CL-O_2_^−·^, FL-OCl^−^ and neutrophil counts, and variation ranges of them were indicated at the upper left in each figure. (**A**) 35 y/o male (n = 39), (**a**) partially expanded view (Day 76–84). (**B**) 35 y/o female (n = 21). (**C**) 43 y/o male (n = 22). (**D**) 42 y/o female (n = 24). (**E**) 52 y/o male (n = 25). (**F**) 54 y/o female (n = 63), partially expanded view (**a**): Day 154–159, (**b**): Day 189–194, (**c**): Day 175–183.

Figure 4 shows scatter diagrams of day-to-day fluctuations of CL-O_2_^−·^ (**○** solid line), FL-OCl^−^ (▲ dashed line), neutrophil count (● dotted line) and CRP (■ solid line) in the six healthy volunteers. Day-to-day dynamics varied largely among individuals and each of them has the unique mean value and variance different from each other. Their coefficients variation (CVs) were as follows, CL-O_2_^−·^: 0.15–0.38, FL-OCl^−^: 0.15–0.43, neutrophil count: 0.12–0.28. Each volunteer’s day-to-day O_2_^−·^ and OCl^−^ values fluctuated within a certain range and from time to time they exerted large fluctuations. Large fluctuations almost always coincided with large changes in the neutrophil count (Fig. 4). Therefore, we analyzed correlations of these values. We denote the correlation coefficient of A and B as Corr(A, B). In most cases Corr(CL-O_2_^−·^, neutrophil count) were higher than Corr(FL-OCl^−^, neutrophil count), although in varying degrees (Table 3 left). There were two uncorrelated cases (Table 3 left, the 43 y/o male and the 52 y/o male, 0 <|R| ≤ 0.2). In the subsequent sections, we further analyzed large signal fluctuations and other signal features in relation to the CRP level, a conventional marker of inflammation, and the questionnaires obtained from the volunteers.

**Table 3:**
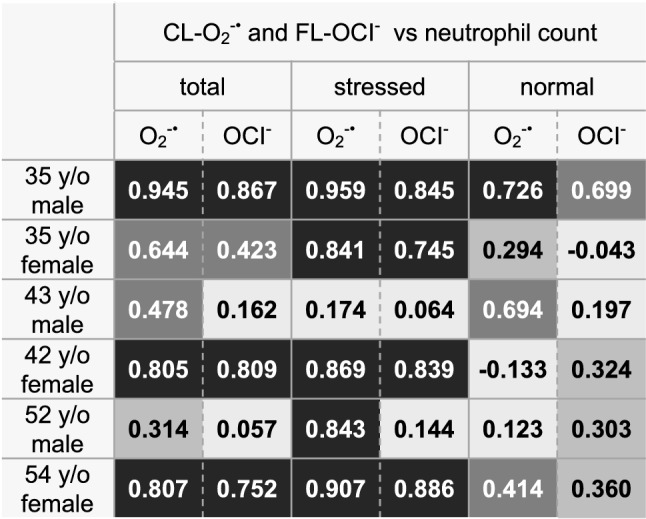
Correlations between CL-O_2_^−·^ and the neutrophil count, and those between FL-OCl^−^ and the neutrophil count.

Left: Correlation coefficients of CL-O_2_^−·^ and FL-OCl^−^ with the neutrophil count in the total data range.

Middle: Correlation coefficients of CL-O_2_^−·^ and FL-OCl^−^ with the neutrophil count in the data range with stressed conditions, i.e., outside the normal/healthy range.

Right: Correlation coefficients of CL-O_2_^−·^ and FL-OCl^−^ with the neutrophil count in the normal data range.

### Effects of acute pharyngitis and gastroenteritis on O_2_^−·^ and OCl^−^ in blood: a case study with a 35 y/o male

On Day 78 of the experiment, the 35 y/o male in the volunteers reported symptoms of a mild sore throat in the questionnaire, but at that time he did not have any fever or other symptoms and looked just normal. O_2_^−·^ and OCl^−^ of that day showed remarkably high values (Fig. 4A and partially expanded view a, arrowhead). On that day a slight amount of CRP was detected in his blood (1 mg/L) and his neutrophil count was more than twice as high as that of the day before. In the evening of that day, when his symptoms became worsened considerably, he had to see the doctor, who diagnosed him with acute pharyngitis and prescribed an antibiotic. The CRP peaked in a day or two after the questionnaire response (Fig. 4A,a, Day 79 and 80), and then decreased (Fig. 4A,a, Day 83). CL-O_2_^−·^, FL-OCl^−^ and the neutrophil count showed the same behavior as CRP, whereas they peaked one day earlier than the CRP peak (Fig. 4A,a, arrowhead). Actually, the same phenomenon had already been observed with this male before this experiment (not included in this study of day-to-day variations). He had acute pharyngitis for 5 days in total. From comparing the data of those five days (n = 5) with the rest of the data in the normal range (the days when his physical condition was normal in his questionnaire, n = 29 with the second illness described below excluded), it was revealed that CL-O_2_^−·^, FL-OCl^−^ and neutrophil count increased significantly (CL-O_2_^−·^ and the neutrophil count: P < 0.01, FL-OCl^−^: P = 0.017).

Then, after Day 133, he kept reporting stomachache in the questionnaire. On Day 154, he was diagnosed with gastroenteritis at clinic and an H2-blocker was prescribed. On Day 155 and 157, he took the medicine. Comparison of the data of these seven days (Day 133- 157, n = 7) and the normal range of data (n = 29) revealed that CL-O_2_^−·^, FL-OCl^−^ and neutrophil count increased significantly (all: P < 0.01).

### Effects of physical activity and viral infection on O_2_^−·^ and OCl^−^ in blood: a case study with a 54 y/o female

She likes to walk as exercise and she had kept recording her daily walk steps with her pedometer. Her daily steps were 6195 on average for the past two years. On some holidays and weekends, she goes on trips and walks 1.5 to 3 times more than usual. On Day 153, she walked 17,475 steps; 8695 steps on Day 154, 12,267 steps on Day 188, and 11,834 steps on Day 189. The obtained data showed that she had remarkably high O_2_^−·^ and OCl^−^ in blood on Days 155 and 190, i.e., on the following days after the long walking trips (Fig. 4F and partially expanded views a, b, arrowheads). On both days, the neutrophil count increased around 1.5–2 times more than her ordinary days (Fig. 4F,a: Day 155, b: Day 190). The CRP peaked on the following day and decreased afterwards. In her case, again, CL-O_2_^−·^, FL-OCl^−^ and the neutrophil count increased earlier than CRP, just like the case of the 35 y/o male with acute pharyngitis (Fig. 4F,a,b).

According to her questionnaire, during her experiment she also had two episodes of virus infection. At one of the two episodes, on Day 172, she was diagnosed with influenza at clinic and stayed in bed for the next four days. After recovery (Day176–179, n = 4), she had remarkably low CL-O_2_^−·^ (Fig. 4F and partially expanded view c, asterisk). The neutrophil count decreased to lower than 50% of the average, and CRP was detected (4 mg/L). Then, the neutrophil count and CL-O_2_^−·^ returned to around the normal, and the CRP level decreased (Fig. 4F,c). Comparison of the data of these four days and the data of the normal days (when her physical conditions did not indicate any problems in the questionnaire, n = 47) revealed that there were significant decreases in CL-O_2_^−·^ and the neutrophil count (P < 0.01), whereas FL-OCl^−^ remained in a tendency to decrease.

Her report about the other incident of virus infection in her questionnaire shows that, on Day 88, she had diarrhea and vomiting. Comparison of the data of five days following the infection (Day 91–94: n = 4) and the normal data (n = 47) revealed that there were significant decreases in CL-O_2_^−·^, FL-OCl^−^ and the neutrophil count (CL-O_2_^−·^: P < 0.01, FL-OCl^−^: P = 0.05, the neutrophil count: P = 0.04).

### Effects of seasonal pollen allergy on O_2_^−·^ and OCl^−^ in blood: a case study with a 35 y/o female, a 42 y/o female, and a 43 y/o male

In their questionnaires, the three volunteers reported that they had symptoms of seasonal pollen allergy multiple times (the 35 y/o female after Day 93 in Fig. 4B, the 42 y/o female after Day 60 in Fig. 4D and the 43 y/o male after Day 81 in Fig. 4C). During the presence of allergy symptoms, all three of them had somewhat different values of CL-O_2_^−·^, FL-OCl^−^ and the neutrophil count. In other words, variable ranges of CL-O_2_^−·^ and FL-OCl^−^ became larger (Fig. 4B–D). Therefore, CL-O_2_^−·^, FL-OCl^−^ and the neutrophil count of these volunteers were compared with or without the symptoms.

At the time when the 42 y/o female became aware of the allergy symptoms (Day 60 in Fig. 4D), FL-OCl^−^ had already increased above her normal level (P < 0.01) while CL-O_2_^−·^ and the neutrophil count only briefly increased and then returned to her normal ranges (CL-O_2_^−·^: P = 0.46, the neutrophil count: P = 0.14). In the case of the 35 y/o female (Fig. 4B), FL-OCl^−^ slowly increased over 2 weeks (from Day 93 to Day 107) and a slight amount of CRP was detected (1–2 mg/L). On the other hand, CL-O_2_^−·^ and the neutrophil count stayed within or near her normal ranges during this period. On Day 108 she was diagnosed with cough-variant asthma at clinic and symptom-relieving drugs were prescribed. On Day 114 CL-O_2_^−·^ and the neutrophil count became remarkably lower, and FL-OCl returned near normal due to the medications. But her symptoms did not disappear, and CRP was detected at 4 mg/L (Fig. 4B). On Day 121 CRP was not detected and her symptoms were improved.

### A case study with a high CRP volunteer

This volunteer is a 52 y/o male and during his experiment we observed CRP on all days. His day-to-day variation data is shown in Fig. 4E. Although his CRP indicates that his liver had been responding to some kind of inflammation in his body, the averages of his CL-O_2_^−·^, FL-OCl^−^ and neutrophil count were almost same levels as those of 42 y/o female who no detected CRP. Therefore, we analyzed Corr(CL-O_2_^−·^, CRP), Corr(FL-OCl^−^, CRP) and Corr(neutrophil count, CRP). It was found that Corr(FL-OCl^−^, CRP) was positive (R = 0.490), while Corr(CL-O_2_^−·^, CRP) and Corr(neutrophil count, CRP) were weakly negative, respectively (R = − 0.210, R = − 0.392).

Next, we considered about the values of CL-O_2_^−·^, FL-OCl^−^ and the neutrophil count on the days when his CRP exceeded the standard value (3 mg/L) (Day 21, 78, 102 and 103). Four days before Day 21 when CRP was 5 mg/L, he had caught a cold. On Day 21 only FL-OCl^−^ was slightly higher than the normal range. Four days before Day78 when CRP was 8 mg/L, he started to have a fever and was diagnosed with a common cold at clinic; however, because the neutrophil count and CL-O_2_^−·^ was low, his data may indicate that he had a more virulent virus infection rather than a common cold. On Day 101 he had abdominal pain. On Day102 his CRP went up to 6 mg/L, and on Day 103, it was still high (4 mg/L). The neutrophil count of these days was not different from his normal range, but both CL-O_2_^−·^ and FL-OCl^−^ were slightly higher.

### Daily variations of O_2_^−·^, OCl^−^ and the neutrophil count in the normal range

So far, we reported about case studies of six volunteers and that most of the time they were in the normal healthy state, although they experienced some illnesses from time to time. We calculated day-to-day variations in their normal range excluding major incidents of illness. Their CVs were as follows: CL-O_2_^−·^ was 0.05–0.18, FL-OCl^−^ was 0.12–0.19, and the neutrophil count was 0.10–0.17. All of them fluctuated in the same range.

Next, since we were not sure about the cause of their day-to-day fluctuations, we analyzed Corr(CL-O_2_^−·^, neutrophil count) and Corr(FL-OCl^−^, neutrophil count) within the normal range only (Table 3, right). The table shows that O_2_^−·^ and OCl^−^ within the normal range were in lower correlation with the neutrophil count than those of the total data range, excluding a few exceptions (O_2_^−·^ and OCl^−^ of the 43 y/o male and OCl^−^ of the 52 y/o male) (Table 3, left and right). As expected, O_2_^−·^ and OCl^−^ in the stressed conditions, i.e., outside the healthy/normal range, were mostly in higher correlation with the neutrophil count than those of the total data range (Table 3, middle, excluding both correlations of 43 y/o male and the OCl^−^ correlation of 35 y/o male).

## Discussion

In “Results”, we have shown the following two important outcomes: (1) a new simultaneous CL/FL monitoring system (CFL-H2200/2400) was confirmed to be optimized for stable blood sample measurements with higher sensitivity for a very long period of time (Figs. 1, 2, Fig. S1). (2) We were able to clarify the fluctuating ranges of O_2_^−·^ and OCl^−^ production in a healthy state and the relationship between the changes in physical condition and their production, which provides insight into the question of whether or not the leukocyte activity can distinguish between more or less unhealthy states and healthy ones.

So far, in the line of our CL/FL simultaneous monitoring project, we have developed three types of systems (Fig. S2, Table 1). Each system is to be used for suitable purposes depending on the characteristics and/or requirements (Table 1). The glass slide-type system is quite sensitive but has some drawbacks for blood sample measurements, i.e., somewhat noisy signals due to air-flow mixing, potential biohazard risks and requirement of skills for air-flow adjustment. To overcome the problems, we contrived a new measurement container, a fluidic-chip type, convenient for blood measurement with the necessary adaptations of the simultaneous CL/FL monitoring optical unit fitted for the fluidic-chip (CFL-H2200/2400). We selected an optical system (excitation from the same side as the detector) that is more suitable for detecting surface-reflected FL rather than transmitted FL. It should also be avoided to collect transmitted FL because of the difficulty to eliminate intense excitation light directly entering PMT through optical filters in addition to the fact that a part of the excitation light as well as FL may potentially be blocked by the absorbing components such as red blood cells, which may worsen signal-to-noise (S/N) ratios. The size of the fluidic-chip, the amount of blood, and the ratio of dilution were carefully examined so that the minimum necessary number of leukocytes should always be existing in the detection field. More specifically, based on the results from isolated neutrophil suspension tests, approximately 5 × 10^2^ cells of neutrophil in the detection field (equal to the LED irradiated field, approximately 10 mm in diameter) were found to be more than enough to give good CL and FL signals. With the actual healthy blood samples (3 µL) after 250-fold dilution, the estimated number of cells in the detection field ranged from 0.78 × 10^3^ to 2.89 × 10^3^, which is definitely more than enough for reliable measurements. Thus, we could determine the optimal conditions for obtaining good enough signals, i.e., 250-fold dilution of 3 µL of blood with the finalized fluidic chip size. These efforts had led us to realize the new system with only a small amount of self-collected blood. The CFL-H2200/2400 system turned out to be almost three times as sensitive as the glass slide-type system. Therefore, the new system was able to measure leukocyte-derived O_2_^−·^ and OCl^−^ for a long time with a greater stability and smaller S/N ratios (Fig. 2, Fig. S4). This is largely attributable to the improvement of stirring efficiency by including a stirrer bar and a stirring space in the fluidic-chip. In addition, separation of the pump section and the measuring section reduced signal fluctuations, which contributed to a remarkable improvement in the S/N ratios. Because of the stability of fluorescein molecules formed after APF reaction with OCl^−^, the value of FL-OCl^−^ was changed from the AUC in the previous report^9^ to FL_MAX_–FL_BASE POINT_ (‘a−b’ in Fig. S3) allowing more accurate assessment.

With this newly developed system, we were able to reveal daily intensity variations of the O_2_^−·^ signal (= CL-O_2_^−·^) and the OCl^−^ signal (= FL-OCl^−^) in six healthy subjects for over several months (Fig. 4). Each volunteer was found to have its own unique signal levels. The signal levels in the subject’s healthy periods finely fluctuated within the CV of 0.20. When anomalies in its physical conditions occurred, the signals were observed as larger deviations beyond its normal fluctuation range (arrows and asterisks in Fig. 4). These large deviations were mostly correlated with increase or decrease of the neutrophil count. We can notice that, between the two signals, the O_2_^−·^ signal was more correlated with the neutrophil count (Table 3, left and middle). This may be due to the fact that O_2_^−·^ is the primary metabolite when neutrophils were stimulated^6,7^.

Next, the overall –O_2_^−·^ and OCl^−^ signals were compared with their cellular productions. We have previously reported that the O_2_^−·^ and OCl^−^ signals contain information about the cellular capacities for generating O_2_^−·^ and OCl^−^ in addition to the neutrophil count^9^. Therefore, by calculating the values per unit number of neutrophils (1 × 10^3^ cells), i.e., O_2_^−·^ production/unit and OCl^−^ production/unit, we will be able to investigate the cellular ROS production capacities. These cellular parameters were found to show comparable variability with the overall O_2_^−·^ and OCl^−^ signals, but they were less dependent on individuals than the overall O_2_^−·^ and OCl^−^ signals.

Then, we analyzed correlations in these three physical conditions, i.e., Corr(O_2_^−·^, O_2_^−·^ production/unit) and Corr(OCl^−^, OCl^−^ production/unit) in the total data range (Table 4, left), within the normal data only range (Table 4, right) and outside the normal data range (Table 4, middle). The correlation coefficients varied among the volunteers. Excluding the 35 y/o female, Corr(OCl^−^, OCl^−^ productions/unit) were higher than Corr(O_2_^−·^, O_2_^−·^ productions/unit) in the total data range (Table 4, left). In the cases of the 35 y/o male, the 42 y/o female, and the 54 y/o female, all of whom had greater neutrophil count variability due to their physical conditions, Corr(O_2_^−·^, O_2_^−·^ productions/unit) were all zero or very low. Within the normal data only range, Corr(OCl^−^, OCl^−^ production/unit) were higher than Corr(O_2_^−·^, O_2_^−·^ production/unit) in all volunteers (Table 4, right). These results may be reflecting the fact that OCl^−^ is the secondary metabolite^7,15^ in ROS productions and more dependent on cellular responses. Furthermore, in the normal conditions, Corr(O_2_^−·^, O_2_^−·^ production/unit) and (OCl^−^, OCl^−^ production/units) (Table4, right) were higher than Corr(O_2_^−·^, neutrophil counts) and Corr(OCl^−^, neutrophil counts) (Table3, right), which suggests that the fine variability of the O_2_^−·^ and the OCl^−^ signals in the normal conditions were derived from the variability of cellular production capacities of O_2_^−·^ and OCl^−^.

**Table 4:**
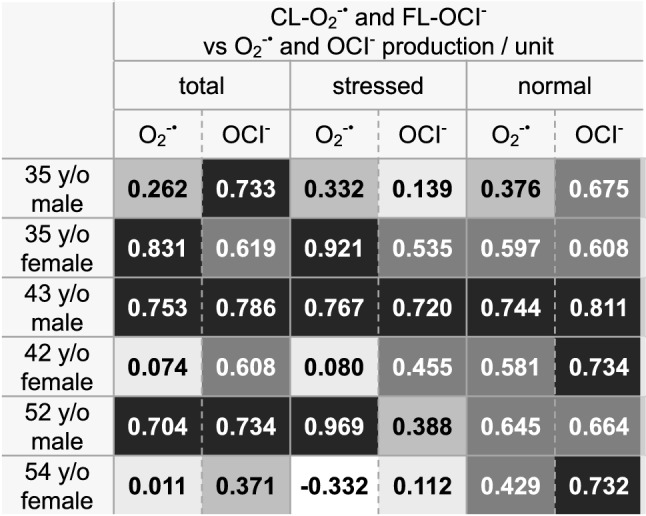
Correlations between CL-O_2_^−·^ and the production capacities of O_2_^−·^, and those between FL-OCl^−^ and the production capacities of OCl^−^

Left: Correlation coefficients of CL-O_2_^−·^ vs the O_2_^−·^ production/unit and FL-OCl^−^ vs the OCl^−^ production/unit in the total data range.

Middle: Correlation coefficients of CL-O_2_^−·^ vs the O_2_^−·^ production/unit and FL-OCl^−^ vs the OCl^−^ production/unit in the data range with stressed conditions.

Right: Correlation coefficients of CL-O_2_^−·^ vs the O_2_^−·^ production/unit and FL-OCl^−^ vs the OCl^−^ production/unit in the normal data range.

**Figure 5:**
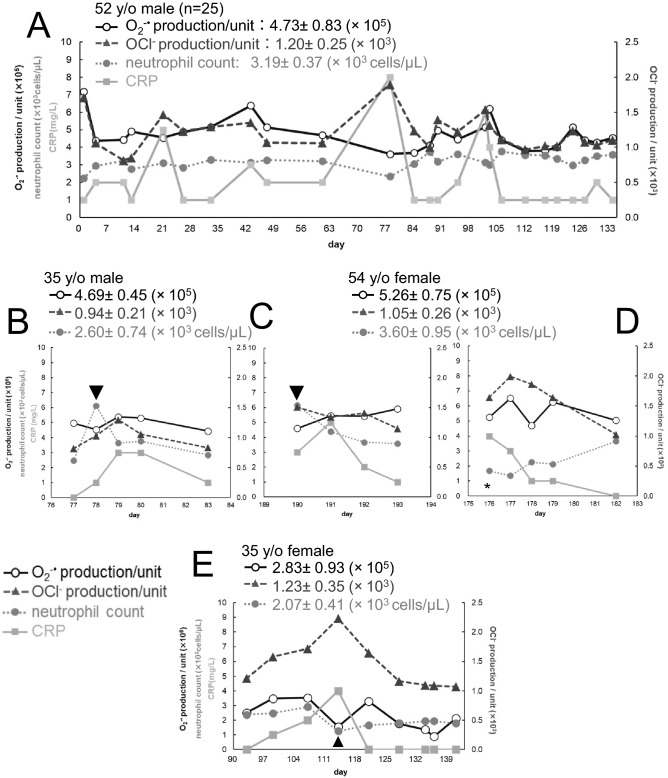
Day-to-day variations of the cellular ROS production capacities. Scatter plots showing the day-to-day fluctuations of the O_2_^−·^ production/unit (○ solid line), the OCl^−^ production/unit (▲ dashed line), the neutrophil counts (● dotted line), and CRP (■ solid line). The left vertical axes indicate the O_2_^−·^ production/unit, the neutrophil counts and CRP; the right vertical axes, the OCl^−^ production/unit. The horizontal axes indicate dates (weekly intervals) during the measurements. The mean values of the O_2_^−·^ production/unit, the OCl^−^ production/unit and the neutrophil counts together with the variation ranges of them were indicated at the upper-left in each figure. (**A**) 52 y/o male (complete data) as an example of six subjects. (**B**) 35 y/o male, partially expanded view (Day 76–84). (**C**) 54 y/o female, partially expanded view (Day 189–194). (**D**) 54 y/o female, partially expanded view (Day 175–183). (**E**) 35 y/o female, partially expanded view (Day 90–143).

We then examined relationships between the cellular ROS production capacities and the volunteers’ physical conditions. Figure 5A shows, as a representative example, scatter diagrams showing the daily variation of the O_2_^−·^ production/unit (**○** solid line), the OCl^−^ production/unit (▲ dashed line), the neutrophil count (● dotted line) and CRP (■ solid line) for the 52 y/o male. We first focused on the secondary response of neutrophils, i.e., the OCl^−^ production/unit. In the above example, when CRP showed high values, the OCl^−^ production/unit also showed high values. By comparing the data of the four days (n = 4) when CRP became more than 4 mg/L and the normal data (n = 21), it was revealed that there was a significant increase in the OCl^−^ production/unit (P < 0.01) while there was no change in the O_2_^−·^ production/unit (P = 0.71). In other cases, when the CRP level was high, independent of the neutrophil count, almost all of them had high levels of the OCl^−^ production/unit on the days of acute pharyngitis (the 35 y/o male, Fig. 5B), excess physical activity/influenza (the 54 y/o female, Fig. 5C,D) and cough variant asthma (the 35 y/o female, Fig. 5E). These cases suggest that the OCl^−^ production/unit is strongly related to inflammation. These were consistent with numerous previous reports that myeloperoxidase (MPO) is associated with inflammation^4–6,16^. More detailed descriptions are as follows. In the case of influenza (the 54 y/o female) and cough variant asthma (the 35 y/o female), although the O_2_^−·^ signal decreased significantly together with the neutrophil count, the OCl^−^ signal did not deviate appreciably from their normal ranges (Fig. 4B,F,c). In these cases, the cellular OCl^−^ production capacity seems to be more or less enhanced. In the case of 35 y/o male with gastroenteritis, although CRP was not detected, a significant (P < 0.01) increase in the OCl^−^ production/unit was also observed (data not shown) suggesting that this volunteer had gastric inflammation due to neutrophil overactivation. When the 35 y/o and the 42 y/o females had symptoms of seasonal pollen allergy, the OCl^−^ production/unit was significantly increased from their normal ranges (the 35 y/o female: P = 0.03, the 42 y/o female: P = 0.02). Also, the OCl^−^ production/unit in the 43 y/o male showed an upward trend. These findings are consistent with the previous reports that patients with allergic rhinitis and allergic asthma released more MPO compared to the controls^17,18^.

We then analyzed the primary response of neutrophils. The O_2_^−·^ production/unit tended to decrease on acute pharyngitis (the 35 y/o male, Fig. 5B, arrowhead) and after the physical activity (the 54 y/o female, Fig. 5C, arrowhead), even though the neutrophil count increased rapidly. Peake and Suzuki reported that exercises induced ROS generations in neutrophils^19^. In our study, the O_2_^−·^ signal, the OCl^−^ signal and the neutrophil count were all increased after exercise (Fig. 4F). Reduction of the O_2_^−·^ production/unit might reflect a possibility that immature neutrophils in the marginal and bone marrow pools were mobilized by neutrophil depletion after bacterial infection or physical activity.

Another important finding in this study is about a temporal relationship of a conventional inflammation marker CRP, and the ROS signals. When measured on consecutive days, large fluctuations in the O_2_^−·^ and OCl^−^ signals were detected earlier than CRP (Fig. 4A,a,F,a,b). Since the initial immune response is neutrophil mobilization followed by ROS productions, which in turn induces production of interleukin (IL)-6 that promote CRP synthesis in the liver^20,21^, ROS and hROS can be regarded as “substrates” of oxidative stress and CRP as “its product” in terms of the previously reported explanation^9^. In a fat-load test of 12 female university students (approval number:H-87, the manuscript under preparation), the production capacities of O_2_^−·^ and OCl^−^ peaked in 2 h after the fat intake and then decreased, whereas the production of IL-6 slowly started to rise in 2 h and continued to rise until stopping the experiment after 6 h (data not shown). These results provide experimental evidence that the increase of OCl^−^ production capacity in blood, specifically, in neutrophils, is followed by IL-6 synthesis in macrophages and then CRP synthesis in the liver.

In addition, the present study suggested that leukocytes activation occurs before appearance of the full-blown symptoms of the diseases; in the case of a 35 y/o male with pharyngitis, a significant increase in ROS production was observed earlier than the symptoms such as fever. This episode clearly shows that the increase in ROS production definitely preceded the symptoms about half a day earlier when he was seemingly healthy. There was also a case, not included in this experiment, where the ROS production of a seemingly healthy female subject with no symptoms at all was several times higher than normal. A few hours later, she suddenly became ill and had to leave work early. In these minor cases in terms of the severity of diseases, it may pose too much burden or healthy people to have their blood drawn very frequently even though it is useful. On the other hand, in spite of the drawback of frequent blood samplings, people prone to chronic diseases, such as diabetics and pre-diabetics, may find it acceptable to have their blood drawn frequently enough for their health checks. In fact, in the case of diabetic nephropathy model rats, data shows that the amount of ROS generated was already higher than control rats at the age of 5 weeks, i.e., before the blood glucose levels increased (data not shown, approval number: HPK-2019-13B, approved by the ethical committee of the Central Research Laboratory, Hamamatsu Photonics K.K.). For future routine practice, the timings and/or frequency of blood sampling would be important for general people to maintain their health while avoiding unnecessary samplings.

The present study confirmed that the ROS signals could detect an imbalanced oxidative state earlier than the conventional indicator in diseases or self-aware symptoms. Further, we think that the individual’s normal values of the ROS signals and their variability might have reflected the balance of oxidation and antioxidation including the effects of neutrophil activity and intrinsic antioxidants, which cannot be accurately explained by the number of neutrophils alone. In addition, information on the cellular ROS production capacities are well contained in the OCl^−^ signal, a secondary metabolite, rather than the O_2_^−·^ signal. These results suggest that this method might be detecting subtle changes in bodily conditions that are not expressed in normal neutrophil count fluctuations. For example, Fig. 4 shows that O_2_^−·^ and OCl^−^ signals were elevated on some days not included in the data taken up in the questionnaire, which might be due to unnoticed physical changes or mental stress^22^. In order to further validate the results of the study, we are now in the process of conducting cross-sectional and longitudinal studies to accumulate data on more than 5000 cases in three and a half years. We plan to establish a reference range by stratum and investigate the possibility of early disease diagnosis, mainly targeting chronic diseases.

Based on the present fluidic-chip system, we are developing a less expensive system that can be easily operated by a non-specialist at various facilities such as citizen centers, drug stores and sports clubs. Such a popular-priced system might be used for point of care testing (POCT) to diagnose inflammatory conditions^23,24^ instead of the traditional marker CRP. Recently, a report showed that neutrophil extracellular traps (abbreviated as NETs) could be used to predict the severity of COVID-19 patients^25^, suggesting that such a system could be applied to bedside monitoring as well as primary screening for patient discovery. Furthermore, in new clinical studies for patients with inflammatory diseases we have recently noticed a tendency that, with progression of the disease, the time lags between the stimulation and the ROS signal elevations somewhat increased, while the ROS signal strengths more or less decreased. We speculate that neutrophils exposed to chronic inflammatory conditions became less responsive to additional stimuli. We are currently trying to elucidate this mechanism. We hope that the information on the peaks and the rising points of ROS signals could be a useful marker for undiagnosable diseases, which might enable us to detect minute changes in bodily conditions more accurately and quickly.

## Supplementary Information

Supplementary Information 1.

Supplementary Video 1.
