# Supplementary material for: Correlation between human health and reactive oxygen species produced in blood: a long-term chemiluminescence and fluorescence analysis
Source: Sci Rep. 2021 Jul 15;11:14545. doi: 10.1038/s41598-021-93887-1 (PMC8282623; doi:10.1038/s41598-021-93887-1)
Supplement: Supplementary file 1 — Supplementary Information 1. [file 41598_2021_93887_MOESM1_ESM.pptx]

## Slide 1
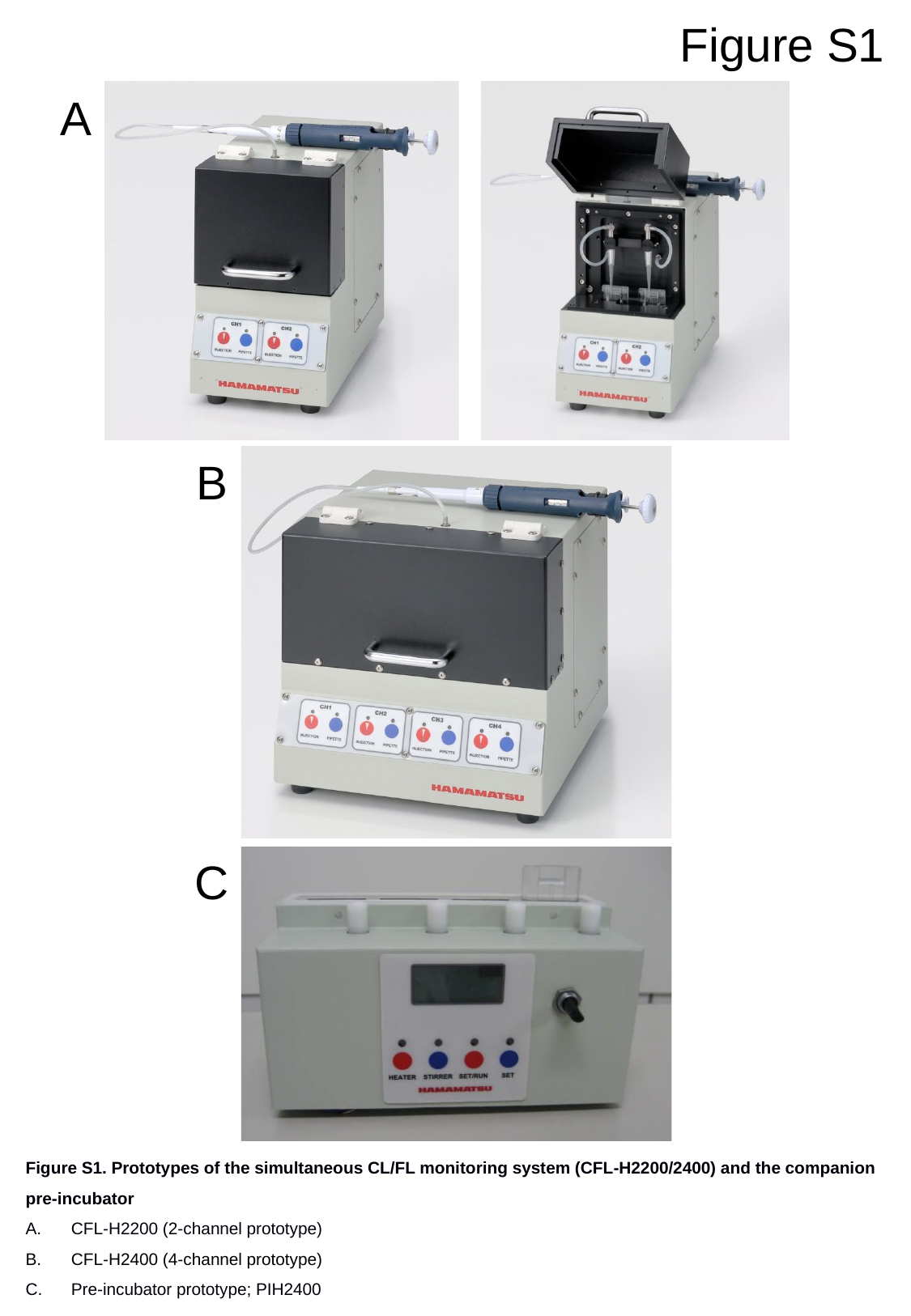

Figure S1
A
B
C
Figure S1. Prototypes of the simultaneous CL/FL monitoring system (CFL-H2200/2400) and the companion pre-incubator
CFL-H2200 (2-channel prototype)
CFL-H2400 (4-channel prototype)
Pre-incubator prototype; PIH2400

## Slide 2
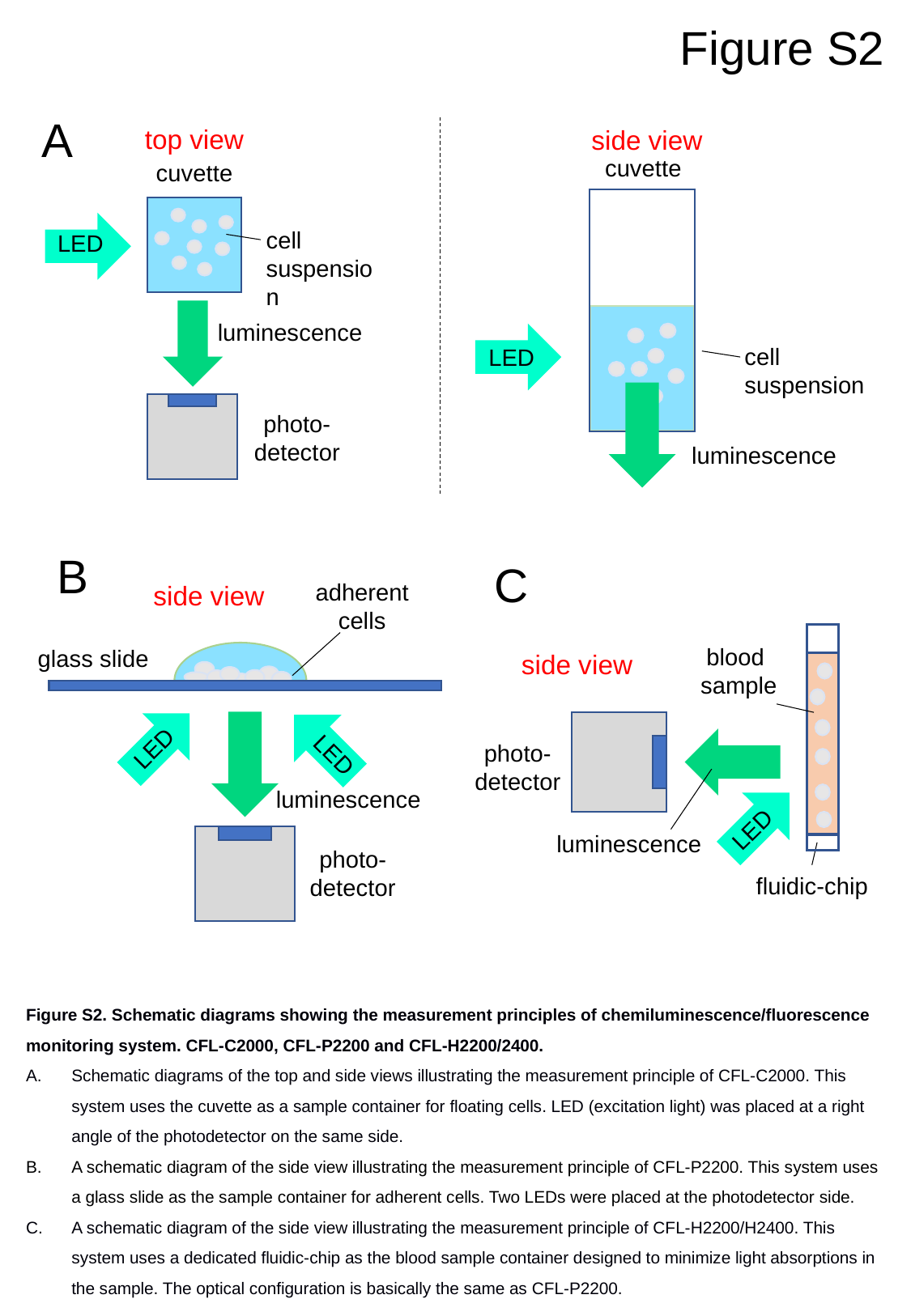

Figure S2
A
top view
cuvette
LED
luminescence
photo-detector
cell
suspension
side view
cuvette
cell suspension
LED
luminescence
B
C
adherent
cells
side view
blood
sample
side view
photo-detector
LED
luminescence
fluidic-chip
glass slide
LED
LED
luminescence
photo-detector
Figure S2. Schematic diagrams showing the measurement principles of chemiluminescence/fluorescence monitoring system. CFL-C2000, CFL-P2200 and CFL-H2200/2400.
Schematic diagrams of the top and side views illustrating the measurement principle of CFL-C2000. This system uses the cuvette as a sample container for floating cells. LED (excitation light) was placed at a right angle of the photodetector on the same side.
A schematic diagram of the side view illustrating the measurement principle of CFL-P2200. This system uses a glass slide as the sample container for adherent cells. Two LEDs were placed at the photodetector side.
A schematic diagram of the side view illustrating the measurement principle of CFL-H2200/H2400. This system uses a dedicated fluidic-chip as the blood sample container designed to minimize light absorptions in the sample. The optical configuration is basically the same as CFL-P2200.

## Slide 3
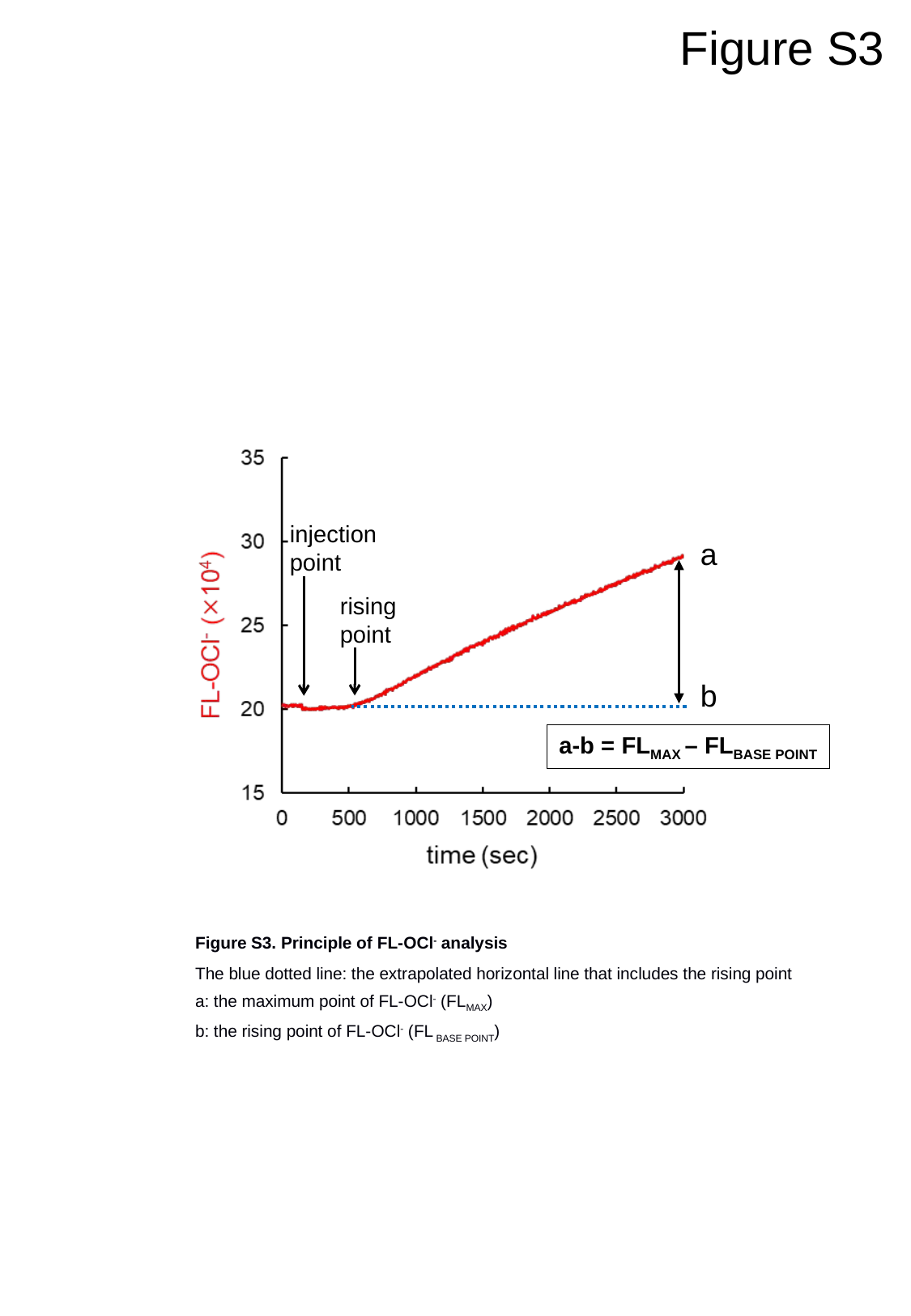

Figure S3
injection point
a
rising point
b
a-b = FLMAX – FLBASE POINT
Figure S3. Principle of FL-OCl- analysis
The blue dotted line: the extrapolated horizontal line that includes the rising point
a: the maximum point of FL-OCl- (FLMAX)
b: the rising point of FL-OCl- (FL BASE POINT)

## Slide 4
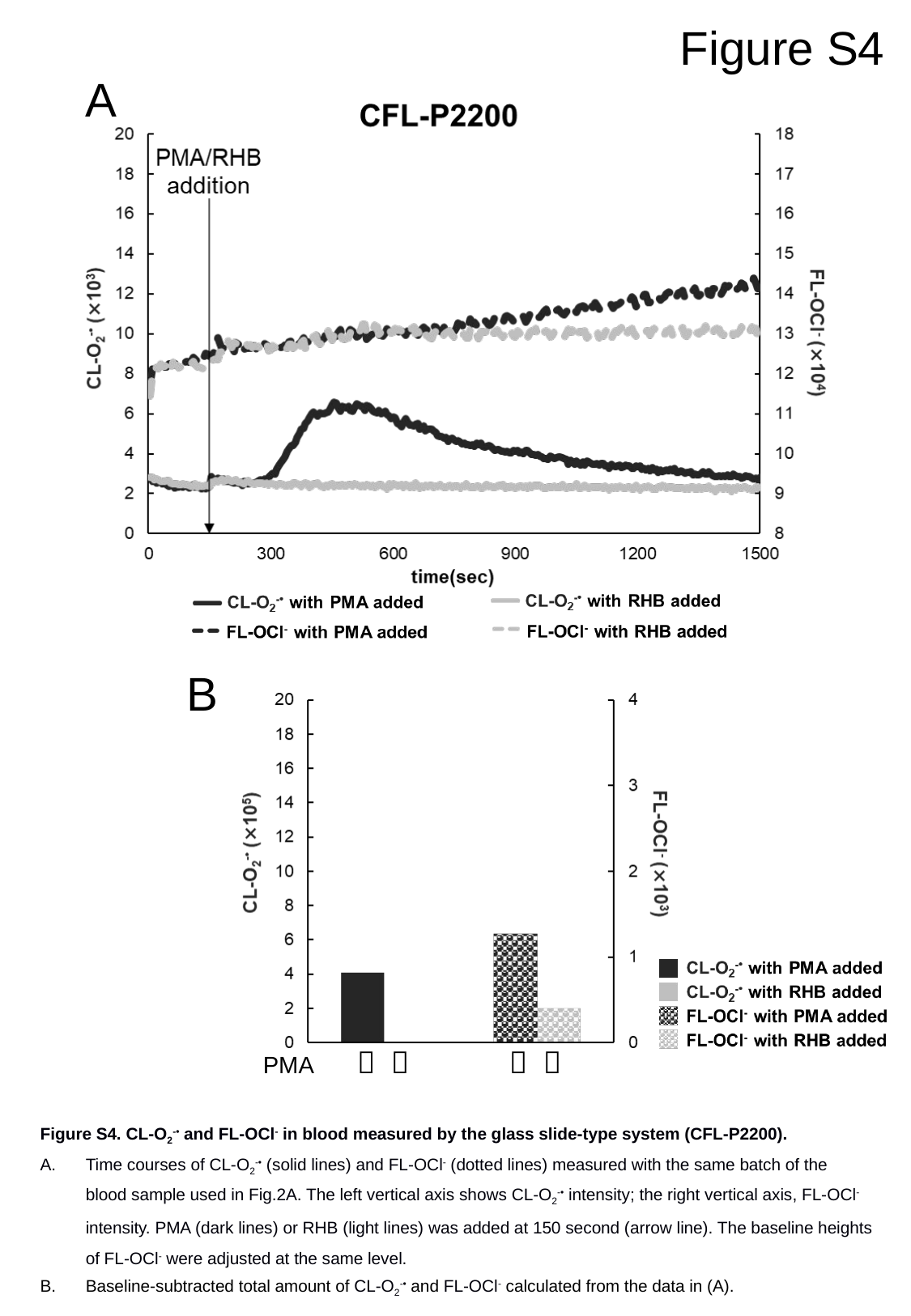

Figure S4
A
B
＋ －
＋ －
PMA
Figure S4. CL-O2-• and FL-OCl- in blood measured by the glass slide-type system (CFL-P2200).
Time courses of CL-O2-• (solid lines) and FL-OCl- (dotted lines) measured with the same batch of the blood sample used in Fig.2A. The left vertical axis shows CL-O2-• intensity; the right vertical axis, FL-OCl- intensity. PMA (dark lines) or RHB (light lines) was added at 150 second (arrow line). The baseline heights of FL-OCl- were adjusted at the same level.
Baseline-subtracted total amount of CL-O2-• and FL-OCl- calculated from the data in (A).

## Slide 5
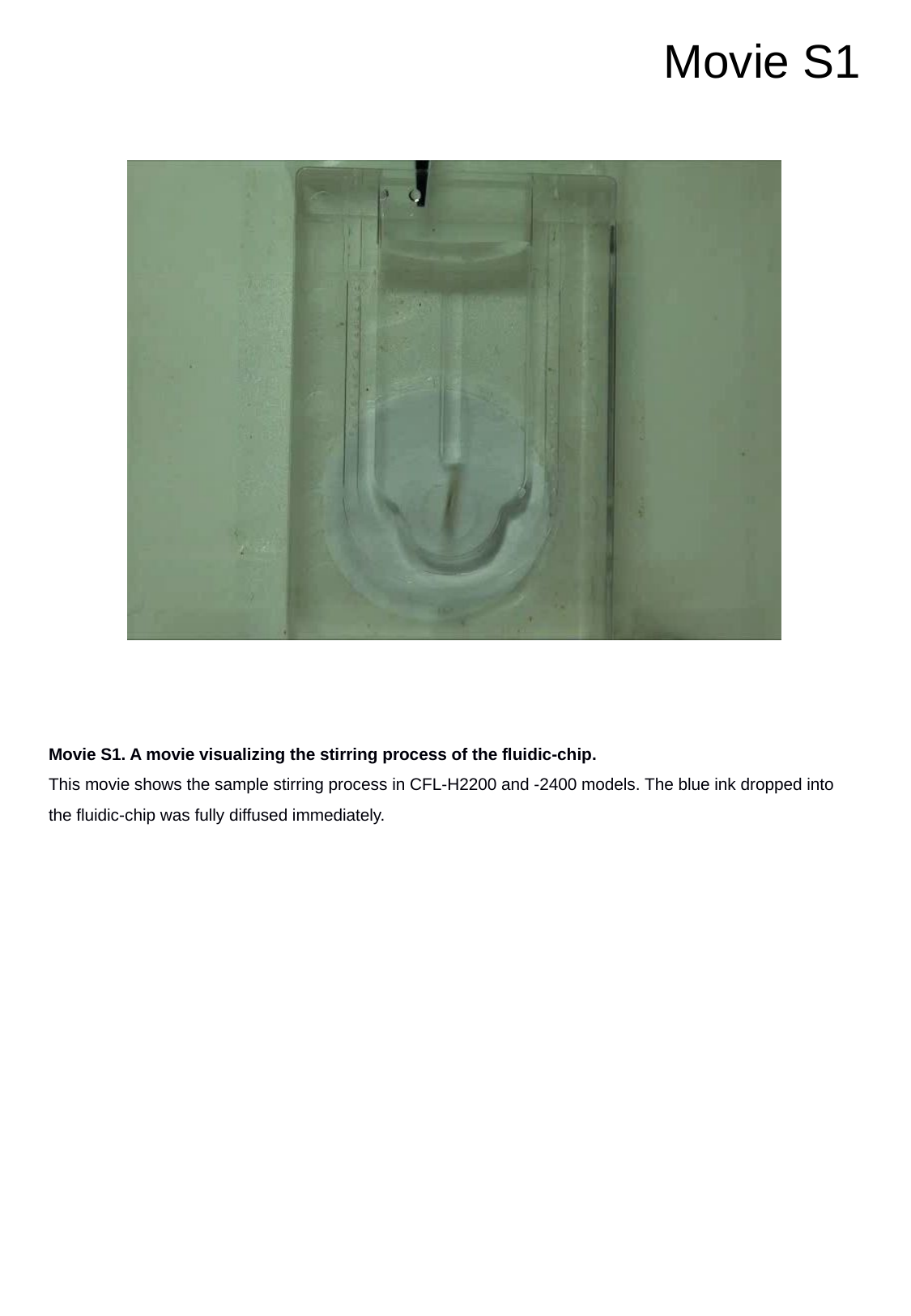

Movie S1
Movie S1. A movie visualizing the stirring process of the fluidic-chip.
This movie shows the sample stirring process in CFL-H2200 and -2400 models. The blue ink dropped into the fluidic-chip was fully diffused immediately.
